# Gender Differences in the Relationship Between Social Communication and Emotion Recognition

**DOI:** 10.1016/j.jaac.2013.08.006

**Published:** 2013-11

**Authors:** Radha Kothari, David Skuse, Justin Wakefield, Nadia Micali

**Affiliations:** University College London

**Keywords:** autism spectrum disorder (ASD), Avon Longitudinal Study of Parents and Children (ALSPAC), emotion recognition, gender, social communication

## Abstract

**Objective:**

To investigate the association between autistic traits and emotion recognition in a large community sample of children using facial and social motion cues, additionally stratifying by gender.

**Method:**

A general population sample of 3,666 children from the Avon Longitudinal Study of Parents and Children (ALSPAC) were assessed on their ability to correctly recognize emotions using the faces subtest of the Diagnostic Analysis of Non-Verbal Accuracy, and the Emotional Triangles Task, a novel test assessing recognition of emotion from social motion cues. Children with autistic-like social communication difficulties, as assessed by the Social Communication Disorders Checklist, were compared with children without such difficulties.

**Results:**

Autistic-like social communication difficulties were associated with poorer recognition of emotion from social motion cues in both genders, but were associated with poorer facial emotion recognition in boys only (odds ratio = 1.9, 95% CI = 1.4, 2.6, *p* = .0001). This finding must be considered in light of lower power to detect differences in girls.

**Conclusions:**

In this community sample of children, greater deficits in social communication skills are associated with poorer discrimination of emotions, implying there may be an underlying continuum of liability to the association between these characteristics. As a similar degree of association was observed in both genders on a novel test of social motion cues, the relatively good performance of girls on the more familiar task of facial emotion discrimination may be due to compensatory mechanisms. Our study might indicate the existence of a cognitive process by which girls with underlying autistic traits can compensate for their covert deficits in emotion recognition, although this would require further investigation.

Autism spectrum disorders (ASD) are characterized by impaired social reciprocity and communication, together with restricted and repetitive behaviors.^1^ More prevalent in males than in females,^2^ the average ratio is 4:1; increasing to 10:1 for a diagnosis of high-functioning autism.^3^ This gender bias may have a biological, cause such as elevated exposure to fetal testosterone or mutations on either the X or Y chromosomes.^4^, ^5^ Alternatively, current diagnostic criteria may not adequately reflect the ASD phenotype in females, resulting in a diagnostic bias toward identification of males. Recent studies suggest that girls adapt better to traits associated with ASD, rendering those deficits less apparent in their day-to-day interactions.^6^, ^7^ Research investigating the prevalence of autistic traits in a general population sample of school-children found a male:female ratio of 2.1:1 on the basis of parent ratings but a ratio of 6:1 when using teacher ratings.^8^ A similar phenomenon was observed in children and adolescents with diagnosed ASD; teachers, but not parents, reported more severe psychopathology in males than in females.^9^ These studies suggest that there is systematic observer bias in recognizing autistic traits. In addition, a recent study has found that affected girls are less likely to be diagnosed with ASD, unless they present with additional difficulties such as low cognitive ability or behavioral problems.^6^

Autistic traits are continuously distributed in the general population, with the most severe end of the continuum being associated with clinical recognition.^8^, ^10^, ^11^, ^12^ Given this continuous distribution, Constantino *et al.* suggest that the cut-offs used for research purposes are arbitrary.^11^ Autistic traits, both within the normal range and at the extreme end of this continuum, appear to share a common etiology.^13^ Research into behavioral traits within the general population may be helpful in understanding ASD as a clinical disorder. Our study was predicated on the observation that ASD-associated cognitive deficits, such as weak central coherence and poor emotion recognition, have recently been found in general population samples that manifest autistic-like behaviors.^14^, ^15^

Deficits in emotion recognition have consistently been associated with clinically diagnosed ASD.^16^, ^17^, ^18^, ^19^, ^20^, ^21^, ^22^, ^23^ In this study, we investigated the association between autistic-type traits and emotion recognition, as well as possible gender differences in this association, in the Avon Longitudinal Study of Parents and Children (ALSPAC), a large population-based cohort in the United Kingdom. In addition, we investigated whether this association would extend to the recognition of emotion from 2 modalities, namely, facial cues and social motion cues. Based on the findings of previous studies, we first hypothesized that children identified from population screening as having poor social communication skills, with behavior traits characteristic of ASD,^24^ would show poorer performance in the recognition of emotion than children without such behavioral characteristics. Because of the accepted gender differences associated with social emotion recognition^25^ and ASD^4^ we analyzed the performance of boys and girls separately as well as together, predicting that when comparing boys high in social communication difficulties to boys without such difficulties, deficits in emotion recognition would be more substantial than when making the same comparison in girls.

## Method

### Participants

The Avon Longitudinal Study of Parents and Children (ALSPAC) is a transgenerational and longitudinal population-based cohort of women (recruited during pregnancy) and the child with whom they were pregnant at the time. Women were eligible if they lived in the study area of Avon during pregnancy and if their expected date of delivery was between April 1, 1991, and December 31, 1992. The initial cohort consisted of 13,867 children, including 199 twin pregnancies.^26^, ^27^ Children and parents have been followed up for the last 21 years through a series of questionnaires, biomedical samples, and physical and behavioral assessments. Behavioral assessments were conducted through clinics to which all parents were invited to bring their children: 7,488 children attended the clinic at 8.5 years of age when the Diagnostic Analysis of Non-Verbal Accuracy was conducted, and 5,844 children attended the clinic at age 13.5 years of age when the Emotional Triangles Task was conducted. Children were eligible for this study if they had completed both of the emotion recognition tasks; and if their parents had completed and returned the Social Communication Disorders Checklist, measuring autistic-like social communication deficits, at age 13.5 years (n = 7,165). The final sample of children with data on all 3 measures was 3,666.

### Measures

#### Social Communication: Social Communication Disorders Checklist (SCDC)

The SCDC^28^ is a 12-item questionnaire that is designed to be completed by parents and that measures social reciprocity and other verbal/nonverbal social traits that are characteristic of ASD. A higher SCDC score is indicative of more deficits in social communication. Studies have found the measure to have good internal consistency (0.93), high test–retest reliability (0.81), and high heritability in both genders (0.74).^28^ In addition, the SCDC has been found to be predictive of autism with a sensitivity of 0.88 and a specificity of 0.91, when using a score of ≥9 out of 24.^10^ Full descriptions of the measure have previously been published.^10^, ^28^

#### Facial Emotion Recognition: Diagnostic Analysis of Non-Verbal Accuracy (DANVA)

Facial emotion recognition was assessed using the faces subtest of the DANVA.^29^ This computerized task measures a child’s ability to recognize emotion from facial cues. Participants were shown photographs of children expressing happiness, sadness, anger, or fear. Higher scores on this task represent more errors or misattributions. A total of 11 binary scores indicating whether children made more (above cut-off) or less (below cut-off) errors/misattributions are considered. These were derived by ALSPAC in collaboration with the creator of the task, Stephen Nowicki. Cut-offs for each of the variables was based on the distribution of results in the whole sample (Table 1).

**Table 1:** Diagnostic Analysis of Non-Verbal Ability Results

Outcome	Binary Cut-off
Happy faces, errors	≥1
Sad faces, errors	≥2
Angry faces, errors	≥4
Fearful faces, errors	≥3
All faces, errors	≥7
All low-intensity faces, errors	≥5
All high-intensity faces, errors	≥3
Faces misattributed as happy	≥4
Faces misattributed as sad	≥3
Faces misattributed as angry	≥2
Faces misattributed as fearful	≥3

Note: Outcome variables with binary cut-offs used.

#### Emotion Recognition From Social Cues: the Emotional Triangles Task

This computerized task measures the participant’s ability to attribute an emotional mental state to nonhuman animate entities. Participants are shown 5-second animations consisting of a triangle and a circle moving around the screen. In 20 of the animations, the triangle moves around in a self-propelled and purposeful manner, which is designed to evoke a mental state attribution of a particular emotion; happy, sad, angry, or scared. In the other 4 animations, the triangle moves in a manner that is designed to look inanimate or “not living.” (Further details of the task, scoring, and animations are provided by Boraston *et al.*^17^) A total of 4 outcome variables, representative of emotion recognition ability for each of the 4 emotions assessed, will be considered here, with a higher score representing better emotion recognition ability.

### Procedure

This study was approved by the ALSPAC Law and Ethics Committee and the Local Research Ethics Committees.

### Data Analysis

All variables were checked for inconsistencies/outliers using tabulations, graphs, and plots. For participants with less than 25% missing data on the SCDC, total scores were calculated using pro-rating. The distribution of variables was inspected for normality. Initially, Spearman rank correlations were conducted to investigate the correlation between the emotion recognition tasks used. The association between social and communication deficits and emotion recognition was then analyzed with linear and logistic regression, using the SCDC score (predictor) as a binary variable according to the recommended cut-off of ≥9, which is predictive of a diagnosis of autism. Scores from the DANVA were not normally distributed, and could not be transformed; therefore, binary variables (as described above) were used, and a logistic regression was conducted to analyze this data. Scores from the Emotional Triangles Task were normally distributed; therefore linear regression was used to analyze these data. The fact that scores on the Emotional Triangles Task are normally distributed, whereas scores on the DANVA are not, is likely to be due to differences in the way that the 2 tasks are scored. Scoring for the Emotional Triangles Task differs to the DANVA in that performance on a combination of “emotional” and “nonemotional” trials are combined to produce a final score for each emotion. In contrast, the DANVA is scored by simply adding up the number of errors/misattributions for each emotion. As a significant number of children made few or no errors, DANVA scores were subject to floor effects, leading to data being positively skewed. Additional linear and logistic regression analyses were conducted to directly compare boys and girls scoring in the top 10th percentile on the SCDC, on both emotion recognition tasks. This was done to compare boys and girls with the most severe social communication difficulties with regard to their emotion recognition ability.

Gender of the child, age of the child at time of each assessment, and the tester conducting the emotion recognition task were included as a priori covariates in minimally adjusted models. Additional confounders that could potentially influence outcomes were adjusted for in fully adjusted models after testing whether these variables met criteria for confounding. All analyses were conducted in boys and girls separately and together, using SPSS 18 (SPSS Inc., Chicago, IL). Because of the number of outcomes within each emotion recognition task, the Bonferroni–Holm procedure was used to adjust for multiple testing.^30^ This procedure uses a stepwise algorithm and is therefore more powerful than the Bonferroni method. Rather than 1 a priori significance level being predetermined, each comparison is assessed sequentially from highest to lowest significance, and the adjusted significance level is determined according to the remaining number of comparisons rather than the total number of comparisons. Only results that were still statistically significant after this adjustment are reported and discussed.

#### Missing Covariate Data

Multiple random imputation was used to deal with missing covariate data. All predictor and outcome variables were used as predictors in the imputation model. Missing data were imputed for marital status, child ethnicity, social class, age of child at time of testing, and parity. All analyses were run on both complete case and imputed datasets for comparison, and differences were negligible. Because complete case analysis is thought to be limited by more chance variation, and because multiple imputation is assumed to correct any bias, only results based on multiple imputation are presented.

#### Attrition

Attrition, that is, children who did not have complete data on all 3 measures (after prorating of SCDC scores), was predicted by child gender, ethnicity, parity and gestational age, marital status of mother during pregnancy, and social class (see Table S1, available online). These variables were included as confounders in the fully adjusted models accordingly.

## Results

### Sociodemographic Data

The sociodemographic data of children included in this study, in comparison to the sociodemographic data of the whole ALSPAC sample, can be found in Table S1, available online. Children for whom data were available were more likely to be white, to have mothers who were married during pregnancy, and to have parents in the higher nonmanual category for social class.

### Correlation of Emotion Recognition Tasks

The vast majority of outcomes from the DANVA and the Emotional Triangles Task were significantly correlated, albeit with low correlation coefficients (see Table S2, available online).

### Gender Differences

Independent-samples t tests were used to compare mean SCDC scores across gender. The mean score in boys (mean = 2.53, SD = 3.69) was significantly higher (more difficulties) than the mean score in girls (mean = 2.25, SD = 3.15) (t_3664_ = 2.40, *p* = 0.02). Results of a χ^2^ analysis also showed that a higher percentage of boys (7.3%) scored above the threshold of ≥9 than did girls (4.7%); **χ**^**2**^(1, N = 3,666 = 10.93, *p* = .001.

χ^2^ Analysis also showed that a higher percentage of boys than girls made errors and misattributions in the recognition of emotion from faces on the DANVA. This was found for the recognition of all faces (25.3% vs. 20.5%), **χ**^**2**^(1, N = 3,666) = 11.69, *p* = .001; all high-intensity faces (22.1% vs. 17.8%), **χ**^**2**^(1, N = 3,666) = 10.61, *p* = .001; all low-intensity faces (22.4% vs. 18.6%), **χ**^**2**^(1, N = 3,666) = 8.25, *p* = .004; happy faces (26.6% vs. 19.1%), **χ**^**2**^(1, N = 3,666) = 29.48, *p* = .0001; sad faces (19.3% vs. 15.7%), **χ**^**2**^(1, N = 3,666) = 7.98, *p* = .005; and angry faces (19.1% vs. 13.1%), **χ**^**2**^(1, N = 3,666) = 24.46, *p* = .0001. In addition, a higher percentage of boys than girls misattributed faces as sad (18.7% vs. 11.4%), **χ**^**2**^(1, N = 3,666) = 37.45, *p* = .0001.

Independent-sample t tests were used to compare mean scores on the Emotional Triangles Task across gender. Surprisingly, boys had higher mean scores (indicative of better emotion recognition) than girls in the angry (mean = 2.76, SD = 1.40 vs. mean = 2.47, SD = 1.43, t_3664_ = 6.07, *p* = .0001) and scared (mean = 2.43, SD = 1.46 vs. mean = 1.91, SD = 1.48, t_3664_ = 10.68, *p* = .0001) conditions, but lower mean scores than girls (indicative of worse emotion recognition) in the sad condition (mean = 1.50, SD = 1.25 vs. mean = 1.61, SD = 1.23, t_3664_ = −2.67, *p* = .01) (Table 2).

**Table 2:** Children’s Scores on the Social Communication Disorders Checklist (SCDC), the Diagnostic Analysis of Non-Verbal Analysis (DANVA), and the Emotional Triangles Task.

	All	Boys	Girls	*p*-value
Sample Size	3,666	1,796	1,870	—
Social Communication Disorders Checklist				
Continuous, m (SD)	2.39 (3.43)	2.53 (3.69)	2.25 (3.15)	0.02^a^
Scoring <9, m (SD)	1.74 (2.18)	1.72 (2.18)	1.76 (2.18)	0.62^a^
Scoring ≥9, m (SD)	12.52 (3.60)	12.71 (3.78)	12.23 (3.32)	0.33^a^
Binary, n (%) scoring ≥9	219 (6.0)	131 (7.3)	88 (4.7)	0.001^b^
Diagnostic analysis of nonverbal accuracy				
Happy faces, n (%) ≥1 error	835 (22.8)	478 (26.6)	357 (19.1)	0.001^b^
Sad faces, n (%) ≥2 errors	640 (17.5)	346 (19.3)	294 (15.7)	0.005^b^
Angry faces, n (%) ≥2 errors	588 (16.0)	343 (19.1)	245 (13.1)	0.0001^b^
Fearful Faces, n (%) ≥2 errors	657 (17.9)	325 (18.1)	332 (17.8)	0.79^b^
All faces, n (%) ≥2 errors	838 (22.9)	454 (25.3)	384 (20.5)	0.001^b^
Low-intensity faces, n (%) ≥2 errors	751 (20.5)	403 (22.4)	348 (18.6)	0.004^b^
High-intensity faces, n (%) ≥2 errors	730 (19.9)	397 (22.1)	333 (17.8)	0.001^b^
Misattributed as happy, n (%) ≥4	498 (13.6)	262 (14.6)	236 (12.6)	0.08^b^
Misattributed as sad, n (%) ≥3	549 (15.0)	335 (18.7)	214 (11.4)	0.0001^b^
Misattributed as angry, n (%) ≥2	415 (11.3)	199 (11.1)	216 (11.6)	0.65^b^
Misattributed as fearful, n (%) ≥2	725 (19.8)	317 (20.7)	354 (18.9)	0.19^b^
Emotional Triangles Task				
Angry, m (SD)	2.61 (1.43)	2.76 (1.40)	2.47 (1.43)	0.0001^a^
Happy, m (SD)	2.10 (1.62)	2.11 (1.71)	2.09 (1.53)	0.73^a^
Sad, m (SD)	1.55 (1.49)	1.50 (1.25)	1.61 (1.23)	0.01^a^
Scared, m (SD)	2.17 (1.49)	2.43 (1.46)	1.91 (1.48)	0.0001^a^

Note: Comparison of whole sample, boys only, and girls only.

a Comparison of mean scores between girls and boys using independent t tests.

b Comparison of number of boys and girls scoring above established thresholds on SCDC and DANVA using χ^2^ tests.

### Emotion Recognition From Facial Cues (DANVA)

#### All Children

A comparison of children scoring high and low on the SCDC (i.e., above or below the threshold of ≥9) showed that children with high SCDC scores had higher odds of making errors in the recognition of all faces (OR = 1.6, 95% CI = 1.3, 2.1; *p* = .0001), all high-intensity faces (OR =1.7, 95% CI = 1.3, 2.2; *p* = .0001), and all-low intensity faces (OR = 1.6, 95% CI = 1.3, 2.1; *p* = .0001) in the minimally adjusted model. All differences remained significant in the fully adjusted model after adjusting for potential confounders (Table 3). Children with high SCDC scores also had higher odds of making errors in the recognition of sad faces in both the minimally (OR = 1.5, 95% CI = 1.1, 1.9; *p* = .01) and fully (OR = 1.6, 95% CI = 1.2, 2.2; *p* = .004) adjusted models, however after adjusting for multiple comparisons, this difference remained significant only in the fully adjusted model. In the minimally adjusted model, children with high SCDC scores had higher odds of making errors in the recognition of angry faces (OR = 1.5, 95% CI = 1.1, 2.0; *p* = .0004); however, in the fully adjusted model, this difference failed to reach statistical significance after adjusting for multiple comparisons (OR =1.5, 95% CI = 1.1, 2.1; *p* = .02). In the minimally adjusted model, children with high SCDC scores had higher odds of making errors in the recognition of fearful faces (OR = 1.6, 95% CI = 1.2, 2.0; *p* = .001) and higher odds of misattributing faces as happy (OR = 1.9, 95% CI = 1.4, 2.5; *p* = .0001) compared to children with low SCDC scores, and these differences remained significant in the fully adjusted model.

**Table 3:** Logistic Regression Analysis of Children’s Facial Emotion Recognition (Diagnostic Analysis of Non-Verbal Ability) Scores (N = 3,666).

	Minimally Adjusted Model^a^	Fully Adjusted Model^b^
	OR (95% CI)	OR (95% CI)
Happy faces (≥1 error)	1.11 (0.86, 1.43)	1.36 (1.00, 1.84) NS
Sad faces (≥2 errors)	1.45 (1.11, 1.90) NS	1.62 (1.18, 2.24)∗∗
Angry faces (≥4 errors)	1.50 (1.14, 1.97)∗∗	1.49 (1.07, 2.09) NS
Fearful faces (≥3 errors)	1.55 (1.20, 2.02)∗∗∗	1.60 (1.16, 2.21)∗∗
All faces (≥7 errors)	1.62 (1.27, 2.07)∗∗∗	1.56 (1.15, 2.12)∗∗
All low-intensity faces (≥5 errors)	1.61 (1.25, 2.07)∗∗∗	1.61 (1.18, 2.20)∗∗
All high-intensity faces (≥3 errors)	1.71 (1.33, 2.19)∗∗∗	1.68 (1.24, 2.29)∗∗∗
Misattributed as happy (≥4)	1.90 (1.44, 2.50)∗∗∗	2.10 (1.50, 2.93)∗∗∗
Misattributed as sad (≥3)	1.23 (0.92, 1.64)	1.24 (0.87, 1.77)
Misattributed as angry (≥2)	1.44 (1.05, 1.98) NS	1.57 (1.08, 2.29) NS
Misattributed as fearful (≥2)	1.24 (0.95, 1.61)	1.19 (0.86, 1.65)

Note: Comparison of children scoring above and below the established threshold on the Social Communication Disorders Checklist. NS = not significant (after adjusting for multiple comparisons using the Bonferroni–Holm method); OR = odds ratio.

a Minimally adjusted model: adjusted for child age, child gender, and tester.

b Fully adjusted model: adjusted for child age, child gender, tester, gestational age, marital status, parity, social class, and child ethnicity.

∗∗ *p* ≤ 0.01, ∗*∗∗p* ≤ 0.001.

#### Girls

A comparison of girls scoring high and low on the SCDC showed that girls scoring high on the SCDC had higher odds of misattributing faces as happy (OR = 1.9, 95% CI = 1.2, 2.9; *p* = .004); however, no statistically significant differences were found in the fully adjusted model after adjusting for multiple comparisons (Table 4).

**Table 4:** Logistic Regression Analysis of Female and Male Children’s Facial Emotion Recognition (Diagnostic Analysis of Non-Verbal Ability).

	Minimally Adjusted Model^a^ OR (95% CI)	Fully Adjusted Model^b^ OR (95% CI)
Females (n = 1,870)		
Happy faces (≥1 error)	0.96 (0.62, 1.49)	1.17 (0.69, 1.99)
Sad faces (≥2 errors)	1.15 (0.73, 1.80)	1.30 (0.75, 2.27)
Angry faces (≥4 errors)	0.98 (0.59, 1.62)	0.64 (0.31, 1.34)
Fearful faces (≥3 errors)	1.47 (0.98, 2.20)	1.47 (0.87, 2.49)
All faces (≥7 errors)	1.23 (0.82, 1.84)	0.97 (0.56, 1.66)
All low-intensity faces (≥5 errors)	1.33 (0.88, 2.01)	1.25 (0.73, 2.13)
All high-intensity faces (≥3 errors)	1.22 (0.80, 1.86)	1.05 (0.60, 1.82)
Misattributed as happy (≥4)	1.88 (1.22, 2.90)∗∗	1.90 (1.08, 3.35) NS
Misattributed as sad (≥3)	0.76 (0.44, 1.34)	0.70 (0.33, 1.48)
Misattributed as angry (≥2)	1.44 (0.89, 2.32)	1.41 (0.77, 2.57)
Misattributed as fearful (≥2)	1.08 (0.71, 1.65)	0.92 (0.53, 1.60)
Males (n = 1,796)		
Happy faces (≥1 error)	1.19 (0.86, 1.65)	1.45 (0.98, 2.13)
Sad faces (≥2 errors)	1.66 (1.18, 2.34)∗∗	1.82 (1.20, 2.74)∗∗
Angry faces (≥4 errors)	1.80 (1.29, 2.52)∗∗∗	2.05 (1.37, 3.08)∗∗∗
Fearful faces (≥3 errors)	1.57 (1.11, 2.22)∗∗	1.69 (1.11, 2.58) NS
All faces (at least 7 errors)	1.92 (1.40, 2.62)∗∗∗	2.00 (1.36, 2.94)∗∗∗
All low-intensity faces (≥5 errors)	1.80 (1.30, 2.48)∗∗∗	1.89 (1.27, 2.80)∗∗
All high-intensity faces (≥3 errors)	2.10 (1.53, 2.88)∗∗∗	2.16 (1.44, 3.25)∗∗∗
Misattributed as happy (≥4)	1.87 (1.31, 2.69)∗∗∗	2.17 (1.42, 3.32)∗∗∗
Misattributed as sad (≥3)	1.49 (1.06, 2.10) NS	1.54 (1.01, 2.35) NS
Misattributed as angry (≥2)	1.45 (0.94, 2.23)	1.73 (1.05, 2.87) NS
Misattributed as fearful (≥2)	1.37 (0.97, 1.93)	1.42 (0.93, 2.16)

Note: Comparison of children scoring above and below the established threshold on the Social Communication Disorders Checklist. NS = not significant (after adjusting for multiple comparisons using the Bonferroni–Holm method); OR = odds ratio.

a Minimally adjusted model: adjusted for child age and tester.

b Fully adjusted model: adjusted for child age, tester, gestational age, marital status, parity, social class, and child ethnicity.

∗∗ *p* ≤ 0.01, ∗*∗∗p* ≤ 0.001.

#### Boys

A comparison of boys scoring high and low on the SCDC showed that boys scoring high on the SCDC had higher odds of making errors in the recognition of all faces (OR = 1.9, 95% CI = 1.4, 2.6; *p* = .0001); all high-intensity faces (OR = 2.1, 95% CI = 1.5, 2.9; *p* = .0001); and all low-intensity faces (OR = 1.8, 95% CI = 1.3, 2.5; *p* = .0001) in the minimally adjusted model. These differences remained significant in the fully adjusted model. A high SCDC score was also associated with errors in the recognition of sad faces (OR = 1.7, 95% CI = 1.2, 2.3; *p* = .004), angry faces (OR = 1.8, 95% CI = 1.3, 2.5; *p* = .001), and fearful faces (OR = 1.6, 95% CI = 1.1, 2.2; *p* = .01), as well as misattributing faces as happy (OR = 1.9, 95% CI = 1.3, 2.7; *p* = .001); however, the association between SCDC scores and anger recognition did not remain statistically significant in the fully adjusted model (Table 4).

### Emotion Recognition From Social Motion Cues

#### All Children

A comparison of children scoring high and low on the SCDC showed that children with high SCDC scores demonstrated poorer performance on the Emotional Triangles Task. In the minimally adjusted model, high SCDC scores were associated with poorer emotion recognition in the happy condition (B = −0.5, 95% CI = −0.7, −0.3; *p* = .0001); this remained significant in the fully adjusted model, in which an association was also observed with poorer recognition in the sad condition (B = −0.3, 95% CI = −0.5, −0.1, *p* = .001) (Table 5).

**Table 5:** Linear Regression Analysis of Children’s Emotion Recognition (Emotional Triangles Task) Scores.

	Minimally Adjusted Model^a^ B (95% CI), R^2^	Fully Adjusted Model^b^ B (95% CI), R^2^
All children (N = 3,666)		
Angry	−0.05 (−0.24, 0.15), 0.01	−0.05 (−0.24, 0.15), 0.02
Happy	−0.51 (−0.73, −0.29)∗∗∗, 0.01	−0.51 (−0.73, −0.29)∗∗∗, 0.01
Sad	−0.28 (−0.46, −0.12)∗∗∗, 0.01	−0.28 (−0.45, −0.11)∗∗∗, 0.01
Scared	−0.17 (−0.37, 0.03), 0.03	−0.17 (−0.37, 0.03), 0.04
Girls (n = 1,870)		
Angry	−0.05 (−0.34, 0.28), 0.001	−0.001 (−0.31, 0.31), 0.01
Happy	−0.47 (−0.80, −0.14)∗∗, 0.01	−0.47 (−0.80, −0.14)∗∗, 0.01
Sad	−0.35 (−0.61, −0.09)∗∗, 0.01	−0.35 (−0.61, −0.08)∗∗, 0.01
Scared	−0.16 (−0.48, 0.16), 0.002	−0.15 (−0.46, 0.17), 0.01
Boys (n = 1,796)		
Angry	−0.06 (−0.31, 0.19), 0.001	−0.06 (−0.31, 0.19), 0.01
Happy	−0.51 (−0.81, −0.21)∗∗, 0.01	−0.55 (−0.85, −0.24)∗∗∗, 0.01
Sad	−0.23 (−0.45, −0.01) NS, 0.01	−0.23 (−0.45, −0.01) NS, 0.01
Scared	−0.17 (−0.43, 0.09), 0.002	−0.17 (−0.43, 0.09), 0.01

Note: Comparison of children scoring above and below the established threshold on the Social Communication Disorders Checklist (B coefficients and 95% CI). NS = not significant (after adjusting for multiple comparisons using the Bonferroni–Holm method).

a Minimally adjusted model: adjusted for child age, child gender, and tester.

b Fully adjusted model: adjusted for child age, child gender, tester, gestational age, marital status, parity, social class, and child ethnicity.

∗ *p* ≤ 0.05*, ∗∗p* ≤ 0.01, ∗*∗∗ p* ≤ 0.001.

#### Girls

In the minimally adjusted model, girls with high SCDC scores demonstrated poorer performance than girls with low SCDC scores in the happy (B = −0.5, 95% CI = −0.8, −0.1, *p* = .005) and sad (B = −0.4, 95% CI = −0.6, −0.1, *p* = .01) conditions. Differences remained significant in the fully adjusted model (Table 5).

#### Boys

In the minimally adjusted model, boys with high SCDC scores demonstrated lower scores in the happy condition (B = −0.5, 95% CI = −0.8, −0.2, *p* = .001) of the Emotional Triangles Task than boys with low SCDC scores. These differences remained statistically significant in the fully adjusted model (Table 5).

### Associations Between Social Communication Difficulties, Facial Emotion Recognition, and Emotion Recognition From Social Motion Cues Across Genders

When directly comparing boys and girls scoring in the top 10th percentile on the SCDC, girls demonstrated lower odds than boys of making errors and misattributions on the DANVA (all faces: OR = 0.42, 95% CI = 0.25, 0.70, *p* = .001). In contrast, girls showed significantly lower scores than boys on the Emotional Triangles Task (for detailed results, see Table S3, available online).

## Discussion

The aim of this study was to investigate, in a large community sample of children, whether emotion recognition difficulties are associated with autistic-like social communication problems. We used 2 measures, 1 assessing the recognition of emotion from facial cues (DANVA) and the other assessing the recognition of emotion from social motion cues (Emotional Triangles Task). In summary, we found that greater social communication difficulties were associated with poorer emotion recognition from both facial cues and social motion cues. Analyzing the performance of boys and girls separately revealed interesting differences. Social communication impairments, within the range typically found in clinically identified cases of autism spectrum disorder, were associated with poorer recognition of emotion from social motion cues in both genders, but were associated with poorer facial emotion recognition in boys only. These findings are discussed in detail below.

When analyzing both boys and girls jointly, higher social communication impairment (SCDC) was associated with more frequent errors in the recognition of emotion from sad and fearful faces, and more frequent misattributions of faces as happy (DANVA). The pattern changed when looking at each gender separately. Boys with high SCDC scores made more errors in the recognition of emotion from sad and angry faces, but no associations were observed in girls. Findings suggest that the established association between ASD and emotion recognition observed in clinical samples^16^, ^17^, ^18^, ^19^, ^20^, ^21^, ^22^, ^23^ is also present, albeit to a lesser degree, in a general population sample of children. These findings also support our hypothesis that facial emotion recognition ability would be relatively more impaired in boys with social communication difficulties than in girls with equivalent behavioral traits. The poorer recognition of negative emotions from facial cues that we observed in this sample, when analyzing the performance of all children or boys alone, is qualitatively similar to deficits observed in clinical groups. Emotion recognition deficits in male adults with ASD, high-functioning individuals with autism, and the parents of children with autism, are particularly marked in terms of negative emotions, including sadness, anger, fear, and disgust. They are less marked in the recognition of happiness.^16^, ^17^, ^18^, ^19^, ^20^, ^21^, ^23^, ^31^

With regard to performance on the Emotional Triangles Task, in the combined population, higher SCDC scores predicted poorer recognition of happiness and sadness from social motion cues. The same pattern of association was observed in girls, whereas in boys high SCDC scores predicted poorer recognition of happiness only. Our findings are consistent with evidence that autistic traits are associated with impaired happiness recognition from nonfacial stimuli such as body movement and vocal cues.^23^

Constantino *et al.* found that the genes that influence autistic-like traits in the general population are the same for both girls and boys, and these investigators suggest that the lower prevalence of autistic traits in girls may be the result of a greater sensitivity to early environmental factors that promote social competency.^11^ Social processes affecting the acquisition of facial emotion processing skills^25^ could be having a protective effect, enabling girls high in autistic-like characteristics to compensate for potential deficits in facial emotion recognition. This hypothesis is supported by the observation that girls in this sample were more accurate in facial emotion recognition in comparison to boys overall, regardless of SCDC scores. The social motion task used in this study was novel, and accurate performance could not have been gained from prior exposure. That the protective mechanism is social, rather than reflecting inherent resilience, is suggested by the observation that girls lacked any advantage over boys in their recognition of novel emotion cues based on movements of inanimate objects. Our findings imply that key features of the autism phenotype, such as impaired facial emotion recognition, which are used to support the clinical assessment of ASD, could be less prominent in girls with equivalent underlying autistic traits. The implications of this are far reaching with regard to the diagnosis of ASD in females, suggesting that more subtle assessment may be required to identify those individuals with difficulties.

Strengths of this study are the use of a large cohort of children, prospective data collection, and the assessment of emotion recognition from 2 different stimuli. In addition, our findings provide further validation for the Emotional Triangles Task, which is a relatively novel measure. This study does, however, have limitations. First, the ALSPAC cohort has been shown to be broadly representative of the Avon area, but not of the United Kingdom as a whole,^27^ limiting the generalizability of findings. Second, mothers who brought their children to clinics were of a higher social class, older, and better educated than those who did not attend.^26^, ^27^ These potential confounders were adjusted for in analyses, but it is still possible that the differences observed may be partially explained by residual confounding. Third, we did not test for interaction between gender and social communication in predicting emotion recognition. Interaction tests typically have limited power, and therefore we chose to use a stratification-by-gender approach to analysis. It is possible that the lack of a significant difference in facial emotion recognition accuracy between girls scoring high and low on the SCDC may have been due to low power, because a smaller proportion of girls than boys scored above the threshold of ≥9. Boys have previously been shown to have mean SCDC scores 30% higher than girls in the ALSPAC cohort.^10^ Alhough it was outside the scope of this study, future research may wish to explore whether using gender-specific thresholds to determine severity might yield different results. Validation of the SCDC in both clinical and population samples does not indicate a need for gender-specific norms, however.^10^, ^28^ It is also worth noting that the effect sizes of differences in the recognition of anger, happiness, and fear were very similar in both genders; and showed a small difference only in the recognition of sadness, on the Emotional Triangles Task. In addition, a direct comparison of boys and girls scoring in the top 10th percentile on the SCDC did show that boys have more trouble with facial emotion recognition, whereas girls had more trouble with recognizing anger and fear from social motion cues. This suggests that there may at least be gender differences in the type of emotion recognition deficits observed in females when compared to males.

To conclude, our findings confirm that more severe problems in social communication autism-like traits are associated with poorer emotion recognition in the general population, suggesting that this association may reflect a continuum of liability, ranging from normal functioning to clinical diagnosis of ASD. Findings from the analysis of boys and girls separately may provide support for the theory that girls adapt better than boys to the impairments associated with ASD, making it less likely that behavioral characteristics in girls will reach the severity required to meet criteria for a clinical diagnosis. Future studies should investigate how female advantages in emotion recognition might affect the presentation of ASD in females, and whether difficulties in emotion recognition from nonfacial stimuli are not sexually dimorphic at the level of neural processing. Performance in facial emotion recognition tasks has been shown to improve throughout childhood and adolescence in typically developing individuals, but not in those with ASD.^32^ This study highlights the importance of investigating the development of emotion recognition skills in girls and boys with ASD separately. To do so may yield important information with regard to the development of successful coping strategies that can be incorporated into management programs for children with ASD.

**Clinical Guidance**

• Impairments in social communication skills are associated with difficulties in emotion recognition from social motion stimuli in girls and boys, but are associated with poorer facial emotion recognition in boys only.
• The lack of association between social communication difficulties and facial emotion recognition in girls suggests that girls might learn to compensate for facial emotion recognition difficulties. This has important implications for current assessment of clinical autism spectrum disorder (ASD) in girls.
• Gender-specific assessment of ASD traits and characteristics might be important to understand the epidemiology of autism spectrum disorder (ASD) and individual treatment needs across genders.

## Figures and Tables

**Table S1:** Comparison of Sociodemographic Data of Whole Avon Longitudinal Study of Parents and Children (ALSPAC) Sample and Sample Included in Study, and Results of Logistic Regression.

Characteristic	Whole ALSPAC Sample (n = 13,761)	Sample Available for Analysis (n = 3,666)	*p* Value
Child gender, male, n (%)	7,579 (51.5)	1,796 (49)	.0001
Child ethnicity, white, n (%)	10,715 (92.5)	3,465 (94.4)	.0001
Parity, multiparous, n (%)	6,473 (55.2)	1,814 (50.3)	.0001
Marital status of mother at enrollment, married, n (%)	9,085 (76.6)	3,056 (84.0)	.0001
Lowest combined parental social class, manual III to manual to V, n (%)	2,247 (19.4)	384 (10.5)	.0001
Gestational age, m (SD)	39.41 (2.27)	39.49 (1.73)	.01

Note: p Values are the outcome of logistic regression analysis of whether socio-demographic factors are predictive of attrition**.** Lowest combined parental social class is a binary value**:** Nonmanual = categories I to III; Manual = manual III to manual V.

**Table S2:** Spearman Rank Correlation of Scores on Emotion Recognition Tasks (N = 3,666)

Diagnostic Analysis of Non-Verbal Accuracy scores	Emotional Triangle Task Scores^a^			
	Angry	Happy	Sad	Scared
All faces, errors	0.08∗∗	−0.06∗∗	−0.08∗∗	−0.08∗∗
All low-intensity faces, errors	−0.08∗∗	−0.05∗∗	−0.07∗∗	−0.07∗∗
All high-intensity faces, errors	0.05∗∗	−0.06∗∗	−0.07∗∗	−0.07∗∗
Happy faces, errors	−0.05∗∗	−0.03	−0.05∗∗	−0.02
Sad faces, errors	−0.06∗∗	−0.04∗	−0.06∗∗	−0.07∗∗
Angry faces, errors	−0.06∗∗	−0.02	−0.03	−0.04∗
Fearful faces, errors	−0.06	−0.06∗	−0.06∗∗	−0.07∗∗
Misattributed as happy	−0.04∗	−0.04∗	−0.06∗	−0.04∗
Misattributed as sad	−0.02	−0.003	−0.03∗	0.002
Misattributed as angry	−0.06∗∗	−0.03	−0.06∗∗	−0.09∗∗
Misattributed as fearful	−0.07∗∗	−0.05∗∗	−0.05∗∗	−0.07∗∗

Note: All variables used continuously.

a Higher score indicates better performance.

∗ *p* ≤ 0.05; ∗*∗p* ≤ 0.01.

**Table S3:** Linear and Logistic Regression Analysis of Emotion Recognition Scores (N = 385; Boys, n = 208; Girls, n = 177)

	Minimally Adjusted Model^a^	Fully Adjusted Model^b^
Diagnostic Analysis of Non-Verbal Accuracy scores	OR (95% CI) *p* Value	OR (95% CI) *p* Value
Happy faces (≥1 error)	0.45 (0.27, 0.76) .003∗∗	0.47 (0.27, 0.80) .01∗∗
Sad faces (≥2 errors)	0.53 (0.31, 0.91) .02∗	0.52 (0.30, 0.91) .02 NS
Angry faces (≥4 errors)	0.25 (0.13, 0.46) .0001∗∗∗	0.22 (0.12, 0.43) .0001∗∗∗
Fearful faces (≥3 errors)	0.81 (0.49, 1.35) .43	0.78 (0.46, 1.31) .34
All faces (≥7 errors)	0.43 (0.26, 0.71) .001∗∗∗	0.42 (0.25, 0.70) .001∗∗∗
All low-intensity faces (≥5 errors)	0.49 (0.29, 0.83) .01∗	0.49 (0.29, 0.83) .01∗∗
All high-intensity faces (at least 3 errors)	0.42 (0.26, 0.69) .001∗∗∗	0.39 (0.23, 0.65) .0001∗∗∗
Misattributed as happy (≥4)	0.83 (0.48, 1.44) .50	0.82 (0.47, 1.44) .49
Misattributed as sad (≥3)	0.25 (0.13, 0.47) .0001∗∗∗	0.23 (0.12, 0.45) .0001∗∗∗
Misattributed as angry (≥2)	0.88 (0.49, 1.60) .68	0.89 (0.49, 1.62) .71
Misattributed as fearful (≥2)	0.68 (0.39, 1.17) .16	0.70 (0.40, 1.23) .21

Emotional Triangles Task scores	B (95% CI) *p* Value	B (95% CI) *p* Value
Angry	−0.44 (−0.73, −0.15).003∗∗	−0.42 (−0.71, −0.13) .01∗∗
Happy	−0.03 (−0.37, 0.32) .0001∗∗∗	−0.05 (−0.40, 0.30) .77
Sad	−0.01 (−0.28, 0.25) .92	−0.03 (−0.30, 0.29) .81
Scared	−0.44 (−0.75, −0.12) .01∗∗	−0.44 (−0.11, −0.12) .01∗∗

Note: Comparison of boys and girls scoring in the top 10% (≥ 7 of 24) on the Social Communication Disorders Checklist (odds ratio [OR] and 95% CI). Diagnostic Analysis of Non-Verbal Accuracy results analyzed using logistic regression: indicates odds of females making a large number of errors/misattributions in comparison to males. Emotional Triangles Task results analyzed using linear regression: indicates higher (better) or lower (worse) scores of females in comparison to males. NS = not significant (after adjusting for multiple comparisons using the Bonferroni–Holm method).

a Minimally adjusted model: adjusted for child age and tester.

b Fully adjusted model: adjusted for child age, tester, gestational age, marital status, parity, social class, and child ethnicity.

∗ *p* ≤ 0.05*, ∗∗p* ≤ 0.01, ∗*∗∗p* ≤ 0.001.
